# Targeting inflammation in heart failure: evolving insights and future directions from randomized clinical trials

**DOI:** 10.1007/s10741-025-10538-7

**Published:** 2025-06-17

**Authors:** Reina Nagasaka, Ellis Kim, Andrew P. Ambrosy, Matthew J. Feinstein

**Affiliations:** 1 The Division of Cardiology, Department of Medicine, Northwestern University Feinberg School of Medicine, Chicago, IL USA; 2 Department of Cardiology, Kaiser Permanente San Francisco Medical Center, San Francisco, CA USA; 3 The Division of Research, Kaiser Permanente Northern California, Pleasanton, CA USA

**Keywords:** Heart failure, Inflammation, Anti-inflammatory therapies, Clinical trials

## Abstract

Heart failure (HF) is a leading cause of cardiovascular morbidity and mortality, with inflammation recognized as a key cause and byproduct. Despite observational studies linking elevated indices of inflammation with HF severity, as well as experimental models highlighting the centrality of inflammation to the pathogenesis of various types of HF, clinical trials of anti-inflammatory therapies in HF have produced inconsistent results. This variability may relate to the substrate included – differences in HF stage and/or clinical phenotype – as well as the mechanisms and target of therapeutics, whether aimed at preventing new-onset HF or treating established disease. This review evaluates clinical trials directly targeting inflammation in HF, with a focus on disease stage and symptomatology. Ultimately, by highlighting the importance of HF staging and the timing of therapeutics in prior inflammation-targeted interventions, we aim to inform more precise targets from a disease substrate perspective when designing trials of inflammation-modulating therapies in HF.

## Introduction

Heart failure (HF) affects over 64 million people worldwide and is one of the leading causes of cardiovascular morbidity and mortality [1]. It is a heterogeneous syndrome characterized by structural and functional abnormalities, leading to impaired ventricular filling and reduced cardiac output. Inflammation is recognized to be a pathophysiological driver of both acute and chronic HF and is associated with a worsening functional cardiac capacity and poor prognosis [2, 3].

Patients with HF have elevated levels of pro-inflammatory cytokines, including interleukin (IL)−1β, IL-6, tumor necrosis factor (TNF)-α, and chemokines, including monocyte chemoattractant protein (MCP)−1, IL-8, macrophage inflammatory protein (MIP)−1α. These cytokines and chemokines are detected both systemically and in the failing myocardium [4–6]. These inflammatory mediators contribute to cardiomyocyte dysfunction, fibroblast activation, and extracellular matrix deposition, leading to disease progression and worsening left ventricular ejection fraction (LVEF) [7]. Myeloperoxidase (MPO), a leukocyte-secreted enzyme that generates reactive oxygen species during inflammatory processes, is also elevated in chronic heart failure patients, further exacerbating myocardial injury [8]. Additionally, higher levels of plasma C-reactive protein (CRP) levels have been associated with increased mortality and morbidity in both acute and chronic HF [9, 10].

In addition to heightened inflammatory activity, inflammation resolution likewise may be impaired in HF. Notably, the production of anti-inflammatory cytokines such as IL-10 is reduced in HF, particularly in patients with HF with reduced ejection fraction (HFrEF) [11]. Similarly, HF patients exhibit a deficiency in specialized pro-resolving lipid mediators, such as resolvin, that are critical for inflammation resolution [12]. This imbalance between inflammation activation and resolution exacerbates endothelial dysfunction, disrupts adrenergic responsiveness, and contributes to adverse myocardial effects, including inotropic dysfunction and sustained remodeling [13].

Despite the central role of inflammation in HF pathogenesis, clinical trials targeting inflammatory pathways have largely failed to achieve meaningful clinical benefits [2]. A recent meta-analysis by Davison et al*.* has shown the promising effects of anti-inflammatory therapies, such as prednisone, anakinra, and colchicine, in reducing mortality, HF readmission, HF exacerbation, and CRP levels in acute HF patients [3]. However, these effects were not consistently observed at the individual trial level, highlighting the variability in outcomes across different therapies and studies. This also raises the possibility that the effect size of these therapies is modest and only discernible when data from multiple studies are pooled in meta-analyses.

While prior reviews have focused on pooling trial results across HF phenotypes or summarizing anti-inflammatory targets, we aimed here to summarize and critique available trial data grouped by substrate (i.e., HF pathophysiology and phenotype) as well as therapeutic target. By contextualizing these trials within the clinical phenotypes and myocardial states at the time of intervention, we offer a substrate- and disease stage-guided framework for evaluating the therapeutic potential of anti-inflammatory strategies in HF.

### Heart failure stages and myocardial states of heart failure

The complexity of HF necessitates a detailed understanding of its classifications to design effective pathophysiology- and clinical phenotype-targeted interventions. Clinical trials of inflammation-targeted therapies in HF often use the New York Heart Association (NYHA) classification to stratify patients based on symptom severity. The NYHA classification categorizes HF into four classes: Class I (no limitations on physical activity) to Class II (slight limitation to physical activity) to the more advanced Class III (marked limitation to physical activity) and Class IV (cannot perform physical activity and symptoms at rest) [14].

While the NYHA classification system is symptom targeted, the American College of Cardiology/American Heart Association (ACC/AHA) staging of HF reflects the pathophysiological progression of the disease, from Stage A (at-risk for HF), Stage B (structural disease without symptoms), Stage C (structural disease with symptoms), to Stage D (refractory HF). For this review, although we will discuss pathophysiology that is relevant to stages, most clinical trial-focused discussion will reference NYHA class of HF as this symptom-focused approach is what is used in trials.

At the tissue level, early HF stages involve extracellular matrix (ECM) remodeling driven by mechanical stress, neurohormonal activation, and inflammatory cytokines. This process activates cardiac fibroblasts and increases matrix protein deposition, which is initially compensatory, but ultimately exacerbates myocardial stiffness and cardiac dysfunction [15]. As HF progresses, persistent ECM remodeling results in extensive fibrosis and irreversible myocardial structural changes, including excessive collagen deposition, architectural disruption, and further impairment of both systolic and diastolic function [16]. For patients in advanced stages of HF, treatment becomes challenging due to the presence of mature myofibroblasts and the activation of fibrotic signaling pathways, such as transforming growth factor (TGF)-β1, which limit the potential for myocardial recovery [17].

Trials targeting at-risk populations or earlier HF stages and/or less advanced symptomatology focus on preventing HF progression, alleviating symptoms, and reducing hospitalizations by addressing the inflammation that drives myocardial injury and remodeling. Conversely, in advanced stages of disease, where fibrosis and permanent damage occur, anti-inflammatory therapies may be less effective. This highlights the importance of tailoring interventions to specific HF stages, with early-stage treatment offering the greatest potential to modify the disease course.

### Clinical trials targeting inflammation in heart failure

**Fig. 1:**
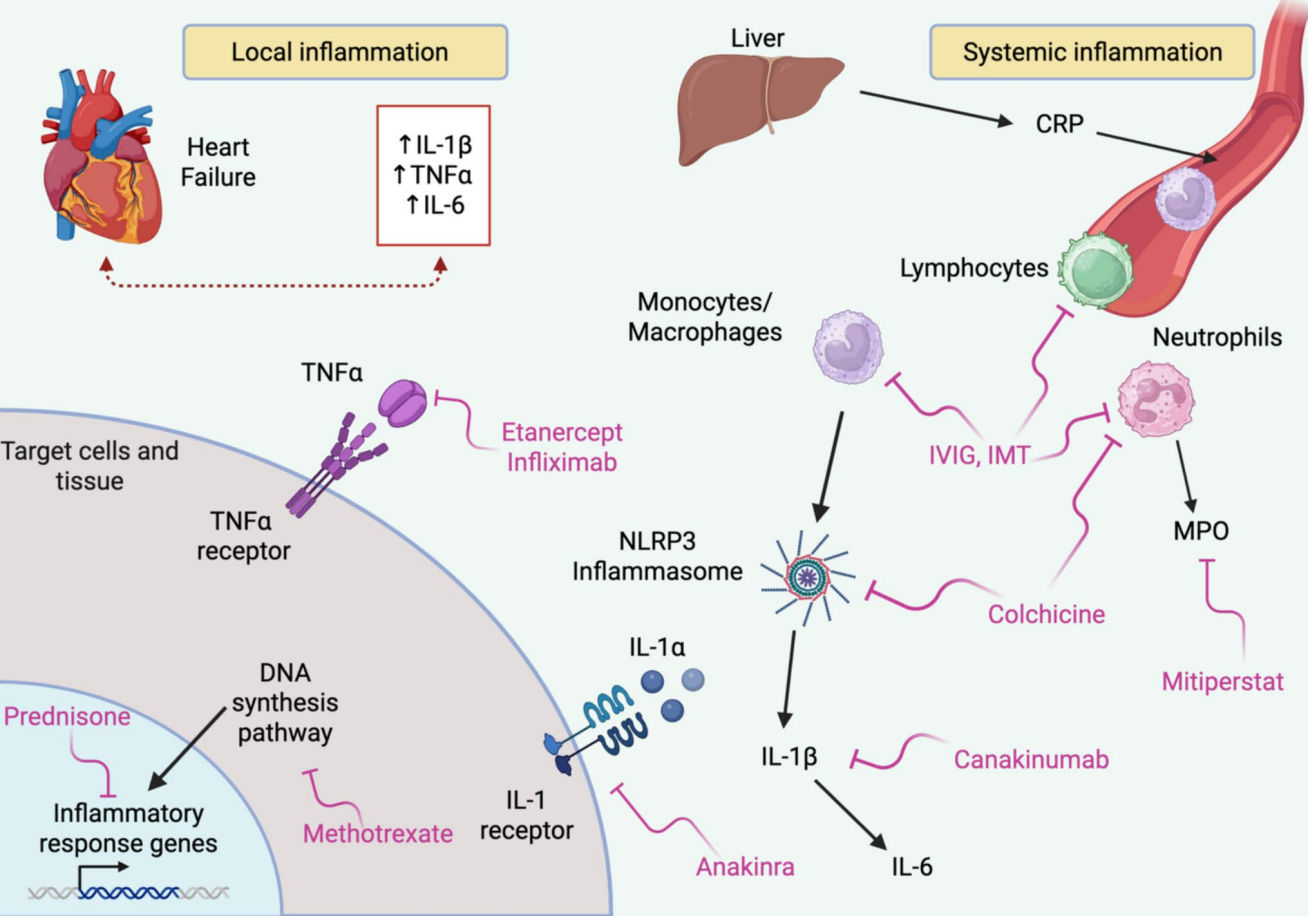
Overview of inflammation pathways targeted in HF. IL = interleukin; TNF = tumor necrosis factor; CRP = C-reactive protein; IVIG = intravenous immunoglobulin; IMT = immunomodulation therapy; NLRP3 = nucleotide-binding domain-like receptor protein 3; MPO = myeloperoxidase

This figure provides a schematic overview of key inflammatory pathways with corresponding targeted drugs that have been or are currently being evaluated in randomized controlled trials involving heart failure (HF) patients. HF is associated with both systemic and local myocardial inflammation, marked by elevated levels of proinflammatory cytokines (i.e., IL-1β, TNFα, and IL-6), chemokines, and enhanced leukocyte recruitment from the peripheral circulation. In response to cardiac injury and neurohormonal activation, neutrophils are among the first immune cells recruited and release MPO, which contributes to oxidative stress and endothelial dysfunction. Recruited monocytes and macrophages further activate the NLRP3 inflammasome, leading to IL-1β production, which in turn induces IL-6 synthesis. CRP, a downstream marker of inflammation, is produced by the liver in response to IL-6 and serves as a clinical biomarker of inflammation. These processes create a positive feedback loop, exacerbating inflammation and driving cardiac remodeling, which accelerates HF progression. Therapeutic strategies targeting these inflammatory pathways, include non-specific IMTs (prednisone, IVIG, device-based IMT, and methotrexate), IL-1 receptor antagonism (anakinra), IL-1β inhibition (canakinumab), NLRP3 inflammasome inhibition (colchicine), MPO inhibition (mitiperstat), and TNFα blockers (etanercept and infliximab) (Fig. 1).

## Discussion

This review summarizes the results of randomized controlled trials (RCTs) investigating anti-inflammatory therapies in HF, with a particular focus on the stage of HF at baseline (also in Table 1, Table 2 and Table 3). The serious adverse events and safety profile across these therapeutic trials are also noted in Table 4. The direct impact of these therapies is examined in the context of patient phenotype and disease severity at the time of treatment.

**Table 1:** Clinical trials with biologics

Drug class across studied HF stage	Trial and study size/characteristics	Study design	Key findings
**TNF**α** inhibitor:** Early HF	Deswal et al., 1999 [18] • *n* = 18 • NYHA III • EF < 35% • TNF > 3.0 pg/mL	Randomized, double-blind, dose-escalation study with 3 cohorts (2 placebo, 4 etanercept): • 1 mg/m^2^ • 4 mg/m^2^ • 10 mg/m^2^	• Improved QOL, 6MWT, and EF at days 1, 2, 7, and 14 for patients receiving 4 or 10 mg/m^2^ • Decrease in circulating levels of biologically active TNF
**TNF**α** inhibitor:** Advanced HF	Bozkurt et al., 2001 [19] • *n* = 47 • NYHA III-IV • EF < 35%	Randomized, double-blind: • Placebo or etanercept 5 mg/m^2^ or 12 mg/m^2^ SC twice weekly for 3 months	Benefits limited to NYHA III: • Dose-dependent increase in EF • Dose-dependent decrease in LVEDV and LVESV • Dose-dependent decrease in LV mass (not statistically significant) • Trend towards significance for improvement in functional status
	RENEWAL 2004 [20] (combined RENAISSANCE & RECOVER) • *n* = 2,048 • NYHA II-IV (predominantly NYHA III and IV) • EF ≤30%	Randomized, double-blind, placebo-controlled: • RENAISSANCE: etanercept 25 mg once weekly vs twice weekly • RECOVER: etanercept 25 mg twice weekly vs three times weekly	• No significant difference in clinical status or combined outcome of death and HHF • Terminated early due to lack of benefit
	ATTACH 2003 [21] • *n* = 150 • NYHA III-IV • EF ≤35%	Randomized, double-blind: • Placebo or infliximab 5 mg/kg or 10 mg/kg at 0, 2, and 6 weeks	• No improvement in clinical status or QOL • 10 mg/kg led to increased risk of death or HHF • Reduced CRP and IL-6 within the first week, then gradual return towards baseline • Reduced serum levels of TNFα after each treatment, then above baseline at all other time points • Improved EF with suppressed CRP and IL-6 levels, however no longer significantly different than placebo group by 28 weeks
**IL-1 antagonism:** No HF established or at-risk for HF	Ikonomidis et al., 2008 [22]: IL-1 receptor antagonism • *n* = 23 • RA with inadequate response to DMARDs • Excluded known or suspected CAD	• Acute: double-blind with crossover trial with placebo or anakinra 150 mg then crossover at 48 h • Chronic: nonrandomized, placebo or anakinra 150 mg daily for 30 days	• Greater reduction in IL-6 and ET-1 (acute) as well as CRP (chronic) • Increased vascular function and coronary flow after acute and chronic treatment with anakinra • Improved LV functional parameters
	CANTOS 2017 [23]: IL-1β antagonist • *n* = 10,061 • Acute MI or within 30 days • hsCRP ≥2 mg/L • Excluded NYHA IV	Randomized, double-blind: • Placebo vs canakinumab 50 mg vs 150 mg vs 300 mg in 1.5:1:1:1 ratio	• Reduced incidence of primary end point (nonfatal MI, nonfatal stroke, CV death) in 150 mg group • Reduced secondary endpoint (composite of primary end point + hospitalization for unstable angina that led to revascularization) in 150 mg group • Reduced CRP • Canakinumab associated with higher incidence of fatal infection
**IL-1 antagonism**: Early HF	D-HART 2014 [24]: IL-1 receptor antagonism • *n* = 12 • RA • NYHA II-III • EF > 50% • hsCRP > 2 mg/L	Randomized, double-blind: • Placebo or anakinra 100 mg daily followed by crossover after 14 days	• Improved peak V_O2_ correlating with reduction in CRP • Improved ventilatory efficiency • No difference in changes to BNP
	REDHART 2017 [25]: IL-1 receptor antagonism • *n* = 60 • NYHA II-III • EF < 50% with acute HF exacerbation • hsCRP > 2 mg/L	Randomized, double-blind: • Anakinra 100 mg daily for 2 weeks then placebo for 10 weeks vs anakinra 100 mg daily for 12 weeks	• Reduced CRP • Increase in peak V_O2_ for group treated for 12 weeks, however not different when compared to other groups • Trend towards significance for improvement in EF • Improved perceived functional capacity in group treated for 12 weeks
**IL-1 antagonism**: Advanced HF	D-HART2 2018 [26]: IL-1 receptor antagonism • *n* = 31 • NYHA II-III (predominantly NYHA III) • EF > 50% • hsCRP > 2 mg/L	Randomized, double-blind: • Anakinra 100 mg daily for 12 weeks vs placebo in 2:1 ratio	• No difference in peak V_O2_ or V_E_/V_CO2_ • Improved exercise time at 4 and 12 weeks of treatment vs baseline; no between-group difference • Reduced CRP with 4 and 12 weeks of anakinra • Improved NT-proBNP levels in anakinra group compared to baseline
**IL-6 antagonism**: At-risk for HF	RESCUE 2021 [27] • *n* = 264 • Stage 3–5 CKD • hsCRP > 2 mg/L	Randomized, double-blind: • Placebo vs ziltivekimab 7.5 mg vs 15 mg vs 30 mg once every 4 weeks for 24 weeks in 1:1:1:1 ratio	• Significant dose-dependent reduction in CRP • Dose-dependent reductions in fibrinogen, serum amyloid A, haptoglobin, secretory phospholipase A2, and lipoprotein(a)

*HF* heart failure, *TNF* tumor necrosis factor, *IL* interleukin, *NYHA* New York Heart Association, *QOL* quality of life, *6MWT* 6-min walk test, *EF* ejection fraction, *LVEDV* left ventricular end diastolic volume, *LVESV* left ventricular end systolic volume, *HHF* hospitalization for heart failure, *DMARD* drug modifying anti-rheumatic drugs, *CAD* coronary artery disease, *RA* rheumatoid arthritis, *BNP* B-type natriuretic peptide, *CV* cardiovascular, *MI* myocardial infarction, *CRP* C-reactive protein, *hsCRP* high-sensitivity C-reactive protein, *CKD* chronic kidney disease

**Table 2:** Clinical trials with non-biologics

Drug name across studied HF stage	Trial and study size/characteristics	Study design	Key findings
**Methotrexate**: At-risk for HF	CIRT 2018 [28] • *n* = 4,786 • History of MI or multivessel CAD • T2DM or metabolic syndrome Excluded NYHA IV patients but baseline NYHA not disclosed	Randomized, double-blind: • Open-label run-in phase for 5–8 weeks with weekly MTX in sequentially increasing doses from 5 to 10 mg to 15 mg • After run-in, placebo vs MTX 15 mg weekly for 4 months, then increased to 20 mg weekly	• No difference in primary end point (MACE or hospitalization for unstable angina leading to urgent revascularization) • No difference in secondary end point (death from any cause, MACE or any coronary revascularization, HHF) • No change to IL-1β, IL-6, or CRP
**Methotrexate**: Advanced HF	METIS 2009 [29] • • *n* = 45 • • NYHA II-IV (predominantly NYHA III/IV) • • EF < 45% • • ≥1 coronary artery with ≥50% lesion on angiography or revascularization > 4 months ago	Randomized, double-blind: • Placebo vs MTX 7.5 mg weekly for 12 weeks	• No significant difference in 6MWT • NYHA scores improved in 66.7% of MTX group vs 50% in placebo group • No change in CRP
**Colchicine**: At-risk for HF	COLCOT 2019 [30] • • *n* = 4745 • MI within 30 days • Excluded severe HF or EF < 35%	Randomized, double blind: • Placebo vs colchicine 0.5 mg daily	• Significant reduction in primary endpoint (CV death, resuscitated arrest, MI, stroke, or urgent revascularization) • No difference in HHF • Reduction in CRP in both placebo and colchicine group
	Akrami et al., 2021 [31] • *n* = 361 • ACS patients who received PCI or medical therapy	Randomized, double-blind: • Placebo vs colchicine 0.5 mg daily	• Significant lower rate of total MACE in colchicine group • No difference in decompensated HF, death from CV or any causes
**Colchicine**: Early HF	Deftereos et al., 2014 [32] • *n* = 267 • EF ≤40% • Excluded NYHA IV (average NYHA 2.4 ± 0.5)	Randomized, double-blind: • Placebo vs colchicine 0.5 mg twice daily for 6 months	• No difference in NHYA class, death, or HHF • Significant reduction in hsCRP and IL-6
**Colchicine**: Advanced HF	COLICA 2024 [33] • *n* = 278 • Acute HF requiring ≥ 40 mg of intravenous furosemide and NT-proBNP > 900 pg/ml	Randomized, double-blind: • Placebo vs colchicine loading dose 2 mg once followed by 0.5 mg twice daily for 8 weeks	• No difference in NT-proBNP levels • No difference in length of hospitalization for index admission • No difference in death or worsening heart failure events
**Mitiperstat (MPO inhibitor):** Early HF	SATELLITE 2024 [34] • *n* = 41 • NYHA II-IV • EF ≥40% • Elevated BNP or NT-proBNP within 1 year • At least 1 hospitalization due to HF or structural heart disease	Randomized, double-blind: • Placebo vs AZD4831 (mitiperstat) 5 mg daily for 90 days in 1:2 ratio	• Terminated early due to COVID-19 pandemic • MPO-specific activity reduction of 67.5% by day 30 and 53.7% by day 90 • Trend towards significance for KCCQ-OSS • No significance difference in 6MWD, coronary flow velocity reserve, or NT-proBNP
	ENDEAVOR 2025 [35, 36] • *n* = 711 • NYHA II-IV • EF > 40%	Randomized, double-blind: • Placebo vs mitiperstat 2.5 mg vs 5 mg daily for 48 weeks	• No difference in KCCQ-TSS or 6MWD • No difference in MACE, HHF, MI, CV death, or all-cause death

*HF* heart failure, *MTX* methotrexate, *IL* interleukin, *CAD* coronary artery disease, *T2DM* type 2 diabetes mellitus, *MACE* major adverse cardiovascular events, *HHF* hospitalization for heart failure, *MPO* myeloperoxidase, *NT-proBNP* N-terminal pro B-type natriuretic peptide, *KCCQ-OSS* Kansas City Cardiomyopathy Questionnaire, overall summary score, *KCCQ-TSS* Kansas City Cardiomyopathy Questionnaire Total Summary Score, *6MWD* 6-min walking distance, *hsCRP* high-sensitivity C-reactive protein, *MI* myocardial infarction, *ACS* acute coronary syndrome, *PCI* percutaneous coronary intervention

**Table 3:** Clinical trials with non-specific immune modulation (IMT)

Studied HF stage	Trial and study size/characteristics	Study design	Key findings
Early HF	Gullestad et al., 2001 [37] • *n* = 40 • NYHA II-III • EF < 40%	Randomized, double-blind: • Placebo vs IVIG 0.4 g/kg daily for 5 days then 0.4 g/kg monthly for 5 months	• Increased IL-1Ra, IL-10, soluble p55-TNFR, and soluble p75-TNFR, indicating anti-inflammatory effects • Improved EF in both CAD and idiopathic DCM groups • Significant decrease in NT-proBNP levels
	ACCLAIM 2008 [38] • *n* = 2426 • NYHA II-IV (predominantly NYHA II-III) • EF ≤30%	Randomized, double-blind: • Sham treatment vs device-based immunomodulation therapy	• Improved QOL score • No difference in death from any cause of CV hospitalization • No difference in HHF, CV death, composite CV events • Nonsignificant reduction in CRP
Advanced HF	CORTAHF 2024 [39] • *n* = 101 • Acute HF • Baseline NYHA II-IV (predominantly III-IV) • NT-proBNP > 1500 pg/ml • hsCRP > 20 mg/L	Randomized, open-label: • Placebo vs prednisone 40 mg daily for 7 days	• Significant reduction in hsCRP • Reduced 90-day risk of worsening HF or death • Improved QOL

*HF* heart failure, *IVIG* intravenous immunoglobulin, *TNFR* tumor necrosis factor receptor, *CAD* coronary artery disease, *DCM* dilated cardiomyopathy, *QOL* quality of life, *CV* cardiovascular, *CRP* C-reactive protein, *hsCRP* high-sensitivity C-reactive protein

**Table 4:** Summary of serious adverse events in anti-inflammatory HF therapies

Drug class/name	Studied HF stage	Safety and serious adverse events by clinical trial
**TNF**α **inhibitor**	Early HF	Deswal et al., 1999 [18] • No serious AE
	Advanced HF	Bozkurt et al., 2001 [19] • No serious AE
		RENEWAL 2004 [20] (combined RENAISSANCE & RECOVER) • No differences in serious AE across groups
		ATTACH 2003 [21] • Significantly increased risk of death or HHF and greater likelihood of hospitalization for any reason in 10 mg/kg infliximab group • Increased risk of serious infections with infliximab
**IL-1 antagonism**	No HF established or at-risk for HF	Ikonomidis et al., 2008 [22]: IL-1 receptor antagonism • No serious AE
		CANTOS 2017 [23]: IL-1β antagonist • No difference in all-cause mortality • Significantly increased risk of fatal infection/sepsis with canakinumab • Significant dose-dependent increased risk of drug-induced liver injury, leukopenia, neutropenia, or thrombocytopenia with canakinumab • Significantly lower risk of cancer mortality with canakinumab
	Early HF	D-HART 2014 [24]: IL-1 receptor antagonism • No serious AE
		REDHART 2017 [25]: IL-1 receptor antagonism • Decreased risk of mortality or HHF at 24 weeks in 12-week anakinra group
	Advanced HF	DHART-2 2018 [26]: IL-1 receptor antagonism • No serious AE
**IL-6 antagonism (ziltivekimab)**	Early HF	RESCUE 2021 [27] • No serious AE
**Methotrexate**	At-risk for HF	CIRT 2018 [28] • No differences in serious AE across groups • Significantly increased risk of non-basal-cell skin cancer and leukopenia with MTX • Significantly increased AST and ALT levels with MTX
	Advanced HF	METIS 2009 [29] • No differences in serious AE across groups
**Colchicine**	At-risk for HF	COLCOT 2019 [30] • Significantly increased risk of pneumonia with colchicine
		Akrami et al., 2021 [31] • No differences in serious AE across groups
	Early HF	Deftereos et al., 2014 [32] • No differences in serious AE across groups
	Advanced HF	COLICA 2024 [33] • No differences in serious AE across groups
**Mitiperstat (MPO inhibitor)**	Early HF	SATELLITE 2024 [34] • No serious AE
		ENDEAVOR 2025 [35, 36] • No differences in serious AE across groups
**Non-specific immune modulation (IMT)**	Early HF	Gullestad et al., 2001 [37] • No serious AE
		ACCLAIM 2008 [38] • No differences in serious AE across groups
	Advanced HF	CORTAHF 2024 [39] • No differences in serious AE across groups

*AE* adverse event, *HF* heart failure, *TNF* tumor necrosis factor, *HHF* hospitalization for heart failure, *IL* interleukin, *OA* osteoarthritis, *MTX* methotrexate, *AST* aspartate aminotransferase, *ALT* alanine aminotransferase, *MPO* myeloperoxidase

### Anti-TNF-α therapy

TNF-α is an inflammatory cytokine that is over-expressed in HF and associated with poor prognosis. Early studies investigating anti-TNF-α therapies, particularly etanercept, a recombinant soluble TNF-receptor, demonstrated potential benefits in small pilot trials. Deswal et al. (1999) conducted a double-blind, dose escalation study of etanercept in NYHA Class III HF patients, which showed significantly improved LVEF in etanercept group (+ 3.8 ± 2.0% vs −3.8 ± 2.9%, *p* = 0.04) compared to placebo. The 6-min walk distance (6MWD), quality-of-life, and ejection fraction (EF) were also improved in the group treated with higher doses (4 and 10 mg/m^2^) [18]. Similarly, a study by Bozkurt et al. (2001) in NYHA III-IV HF patients demonstrated a dose-dependent improvement (from 5 to 12 mg/m^2^) in LVEF and in LV end-diastolic and end-systolic volumes (*p* = 0.01, *p* = 0.04, *p* = 0.02, respectively) with etanercept, although the benefits were observed only in NYHA III patients [19].

However, larger scale trials have failed to replicate these benefits. The RENEWAL (Randomized Etanercept Worldwide Evaluation) trial, which combined data from the RECOVER and RENAISSANCE trial, included a patient cohort with more advanced HF staging (46% NYHA IIIa, 25% NYHA IIIb, and 4% NYHA IV). The primary endpoint of death or hospitalization for HF (HHF) was not reduced with etanercept, thereby leading to early trial termination of both trials due to lack of benefit [20]. Similarly, the ATTACH (Anti-TNF-α in Congestive Heart Failure) trial investigating infliximab, a chimeric monoclonal TNF-α antibody, in NYHA III-IV patients highlighted concerns about the safety of TNF-α antagonism in advanced HF. Through the 28 weeks of the trial, the high-dose group (10 mg/kg) experienced significantly higher rates of death or HHF [hazard ratio (HR) 2.84 with 95% confidence interval (CI): 1.01–7.97; *p* = 0.043] [21]. Although both infliximab groups showed early reduction in CRP and IL-6 within one week and an improvement in LVEF (+ 3.5% with 5 mg/kg and + 2.1% with 10 mg/kg, *p* = 0.039 for placebo versus both infliximab groups combined), these changes did not persist beyond week 14. By week 28, inflammatory biomarkers and LVEF returned to baseline, which coincided with the upwards trend in adverse events, including mortality and HHF. Given that the last infliximab dose was administered at week 6, this deterioration may reflect a loss of anti-inflammatory effect over time, a rebound inflammatory response following abrupt withdrawal of high-dose immunosuppression, or potential dose-dependent toxicity. Collectively, these large-scale studies suggest that anti-TNF-α therapies may be ineffective or even harmful in advanced HF stages, thus emphasizing the need for clinical phenotyping of HF prior to treatment selection. Although initial studies suggest potential benefits in milder stages (NYHA II–III), the efficacy of TNF-α blockade appears to be lower in more progressed disease, highlighting a narrow therapeutic window in which such interventions may be beneficial.

While these early trials evaluated initial safety of etanercept in HF patients, they were generally small in size, had short duration of follow up, and were not designed to systematically evaluate inflammatory biomarkers and the extent to which these were responsive to therapy. The larger RENEWAL trial was performed in a larger sample of patients and enrolled patients with more advanced HF. While the ATTACH trial had a modest patient size and duration of treatment, it was the only TNF-α inhibitor study that measured inflammatory markers longitudinally. However, the numerical values of baseline CRP levels and magnitude of CRP changes were not reported, limiting the interpretation of infliximab effects. The lack of biomarker data overall in these trials limits our ability to identify the correlation between anti-inflammation and clinical outcomes. This gap underscores the potential importance of integrating therapeutic target-responsive biomarkers in future trials.

### Anti-IL-1 therapy

Targeting the IL-1 pathway, a driver of systemic inflammation, has demonstrated potential in HF therapy across early clinical trials. Ikonomidis et al. (2008) investigated anakinra, a recombinant IL-1α receptor antagonist, in patients with rheumatoid arthritis (RA). Anakinra treatment led to a significant decline in IL-6 (*p* = 0.003) and CRP levels (−80 ± 9% vs −58 ± 6%, *p* = 0.004) and improvements in vascular and LV function, measured by enhanced coronary flow reserve (+ 29 ± 2% vs + 4 ± 2%, *p* < 0.001), aortic distensibility (+ 45 ± 3% vs + 2 ± 2%, *p* < 0.001), and echocardiographic E/Em ratio (−15 ± 1% vs −7 ± 1%, *p* = 0.005) when compared to placebo [22]. This study demonstrated that anakinra can improve cardiovascular function, providing a basis for exploring IL-1 blockade in HF.

In CANTOS (Canakinumab Anti-inflammatory Thrombosis Outcomes Study), an IL-1β monoclonal antibody canakinumab was investigated in post-myocardial infarction (MI) patients with elevated high-sensitivity CRP (hsCRP). Canakinumab reduced the primary endpoint of stroke, nonfatal MI, or cardiovascular death by 15% in the group treated with 150 mg (*p* = 0.021) and HHF with a dose-dependent effect (*p* for trend = 0.025) [23, 40]. At 48 months, treatment with canakinumab led to an hsCRP reduction from baseline that was 26%, 37%, and 41% greater in the 50 mg, 150 mg, and 300 mg group, respectively, compared to placebo group (*p* < 0.001 for all comparisons of median percent change compared to placebo). Notably, *post-hoc* analysis indicated that patients who achieved a reduction in hsCRP with canakinumab had better primary endpoint outcomes (HR 0.62; CI: 0.47–0.81) compared to those with sustained hsCRP levels (HR 1.03; CI: 0.81–1.31). While the baseline HF staging was not reported, the HHF incidence rate was higher among treated patients with a history of HF than treated patients without HF at baseline [40]. Moreover, canakinumab did not lead to greater reductions in HHF in patients with a history of HF at baseline versus those with no history of HF, indicating that its benefit is not specifically enhanced in patients with pre-existing HF. Collectively, these findings underscore the potential of IL-1 inhibition in improving HF outcomes for at-risk patients when systemic inflammation, as measured by inflammatory biomarkers, is adequately targeted.

While these studies investigated HF incidence-related endpoints, the D-HART (Diastolic Heart Failure Anakinra Response Trial) pilot study evaluated RA patients with elevated hsCRP and NYHA II-III heart failure with preserved ejection fraction (HFpEF). Patients treated with anakinra saw improved aerobic capacity, measured by peak oxygen consumption (peak VO_2_), and reduced plasma CRP levels (−6.1 mg/L [−74%], *p* = 0.006), with reductions correlating with improved peak VO_2_ (R =  − 0.60, *p* = 0.002) [24]. The D-HART2 trial showed a significant reduction in CRP (−4.0 mg/L [−65%], *p* = 0.026) and N-terminal pro-B-type natriuretic peptide (NT-proBNP) levels (*p* = 0.022) at 4 weeks in anakinra-treated NYHA II-III HFpEF patients. However, no improvement in peak VO_2_ or ventilatory efficiency was observed [26]. The advanced disease state (NYHA III predominance) in D-HART2 may account for the differences in cardiopulmonary responses compared to the D-HART study, which had an equal distribution of NYHA II and III patients. Additional factors that may have contributed to these observed outcome differences include: the greater degree of CRP reduction in D-HART and the higher prevalence of severe obesity in D-HART2 (60% of participants with body mass index > 40 kg/m^2^ compared to 30%), known to limit aerobic capacity independent of cardiac functioning. Despite the absence of pulmonary fitness improvement in D-HART2, anakinra-treated patients still exhibited reductions in NT-proBNP levels and increases in exercise duration, both linked to favorable HF outcomes. This supports an overall benefit of anakinra in the setting of HFpEF.

In the REDHART (Recently Decompensated Heart Failure Anakinra Response Trial), NYHA II-III HF patients with recently decompensated HF showed significantly reduced CRP levels (−3.4 mg/L from baseline [−66%], *p* = 0.011) and improved peak VO₂ from baseline (*p* = 0.009) with 12 weeks of anakinra treatment. The changes in CRP level correlated with changes in peak VO₂ (*R* = −0.57, *p* = 0.001), supporting the use of IL-1 blockade as a systemic anti-inflammatory strategy in inpatient settings for acute decompensation [25].

Overall, these results support the use of IL-1 inhibition as a promising strategy, particularly in early or acute stages of HF. Anakinra has demonstrated improvements in vascular and cardiopulmonary functioning across multiple studies. The CANTOS trial, with its large sample size and extended follow-up (mean of 3.7 years), also provides evidence supporting canakinumab in reducing cardiovascular events in patients with elevated inflammatory risk. However, limitations should be acknowledged. The REDHART, D-HART, and D-HART2 studies were modest in size and length of study and conducted at a single center, which may limit its generalizability across other clinical settings and patient populations. D-HART also only had one male participant and, thus, was not representative of the demographics of the general population. These limitations underscore the need for large, multicenter trials to validate and extend these results.

### Anti-IL-6 therapy

IL-6, a downstream cytokine of IL-1 signaling, is elevated in HF patients and independently associated with increased risk of mortality and hospitalization [41]. The RESCUE (Trial to Evaluate Reduction in Inflammation in Patients With Advanced Chronic Renal Disease Utilizing Antibody Mediated IL-6 Inhibition) study investigated ziltivekimab, an IL-6 ligand monoclonal antibody, in individuals with chronic kidney disease (CKD), elevated hsCRP, and high cardiovascular risk [27]. After 12 weeks, ziltivekimab produced a significant dose-dependent reduction in the primary outcome of hsCRP (−77% for 7.5 mg, −88% for 15 mg, and −92% for 30 mg, *p* < 0.0001). Dose-dependent reductions were also observed in atherothrombotic biomarkers, including fibrinogen, serum amyloid A, haptoglobin, secretory phospholipase A2, and lipoprotein(a). These anti-inflammatory effects occurred without changing lipid parameters (i.e., total cholesterol to high-density lipoprotein ratio), supporting a mechanism independent of lipid modulation. A post-hoc analysis also demonstrated a significant reduction in the neutrophil-to-lymphocyte ratio, consistent with suppression of myeloid-driven inflammation [42]. RESCUE did not look into the clinical implications of reducing inflammatory and thrombotic biomarkers. Therefore, building upon these results, the ongoing phase 3 ZEUS (Effect of Ziltivekimab vs Placebo on Cardiovascular Outcomes in Participants with Established Atherosclerotic Cardiovascular Disease) trial evaluates the impact of ziltivekimab on MACEs and renal outcomes in a similar CKD with elevated hsCRP patient population [43].

The RESCUE trial data is also one of the first providing evidence of dose-dependent reduction of atherothrombotic markers via IL-6 inhibition. Although the RESCUE trial had a modest size and duration of therapy, ZEUS will address some of these limitations as it will recruit 6,200 patients and include assessments such as baseline LVEF. The strength of these trials is their focus on patients with elevated baseline inflammation, thereby enriching the study population for individuals more likely to benefit from anti-inflammatory therapy. The targeted enrollment of individuals with CKD and high cardiovascular risk is particularly relevant, as these comorbidities are well-established risk factors for HF, particularly HFpEF. Given the modulatory effects of ziltivekimab on atherogenic inflammatory pathways, if the ZEUS trial also confirms similar clinical benefits, IL-6 blockade may emerge as a potential strategy for individuals with similar at-risk profiles for HF.

### Methotrexate

Methotrexate, a folate antagonist with non-specific anti-inflammatory effects mediated by adenosine, has been studied for its potential as a HF therapy. In the METIS (Methotrexate Therapy Effects in the Physical Capacity of Patients with Ischemic Heart Failure) trial, which investigated chronic ischemic HF patients, there was no improvement in the primary endpoint of 6-min walk test with low-dose methotrexate. However, there was a trend towards improvement in NYHA class after 12 weeks, with 66.7% of the methotrexate group showing improvement compared to 50% of the placebo group (*p* = 0.2) [29]. The absence of significant findings in METIS could be attributed to the lack of hsCRP reduction in the treatment group, indicating insufficient inflammation control with methotrexate. Additionally, the predominance of patients in advanced HF stages (72% NYHA Class III/IV in the methotrexate group versus 52% in placebo) likely contributed to the limited therapeutic response, as these patients may have progressed beyond a stage where anti-inflammatory therapy could impact cardiac remodeling.

In the CIRT (Cardiovascular Inflammation Reduction Trial), methotrexate was studied in patients at risk for HF, defined as those with a diagnosis of type 2 diabetes mellitus or metabolic syndrome and a prior MI or multivessel coronary artery disease. Methotrexate did not reduce cardiovascular events or HHF in this population. However, with the exception of NYHA IV exclusion, the trial did not report baseline HF classes, limiting interpretation of symptom severity at enrollment. Moreover, the plasma levels of key inflammatory markers, including IL-1β, IL-6, and hsCRP, were not reduced, suggesting either inadequate anti-inflammatory action by methotrexate or limitations in the effects of methotrexate on cardiac-specific inflammatory mediators [28].

In these trials, the failure of methotrexate to reduce inflammatory biomarkers warrants the need to better investigate its potential effects on HF under conditions where it demonstrates sufficient anti-inflammatory effects. Although the METIS trial data was limited by a small sample size and short 12-week treatment duration, it provided preliminary insight into the potential use of methotrexate in the setting of HF. However, its ability to impact physical capacity in patients may have been constrained by its limited treatment window and the lack of dose-dependent evaluation. Furthermore, the METIS trial did not report baseline inflammatory biomarker levels and exclusively focused on ischemic HF, which may not generalize to other HF etiologies. CIRT may also have been limited by its inclusion of patients with only modest elevations in systemic inflammation (median hsCRP levels of 1.6 mg/L), possibly enrolling patients less likely to benefit from anti-inflammatory therapies. The lack of HF phenotype detailing of baseline NYHA class also limits interpretation, raising concerns that these patients were too advanced in their HF staging to experience benefit. Despite these limitations, major strengths of CIRT include its large cohort of 4,786 patients and its extended median follow-up of 2.3 years, offering valuable longitudinal data on cardiovascular outcomes.

### Colchicine

Colchicine is an anti-inflammatory alkaloid that disrupts microtubule formation and inhibits nucleotide-binding domain-like receptor protein 3 (NLRP3) inflammasome activation, thereby targeting key inflammatory pathways involved in cardiovascular diseases [44]. The COLCOT (Colchicine Cardiovascular Outcomes Trial) study demonstrated significant reduction in the primary endpoint of cardiovascular death, cardiac arrest, MI, stroke, and urgent angina hospitalization (HR 0.77; CI: 0.61–0.96, *p* = 0.02) in post-MI patients treated with colchicine. While HHF was comparable across groups, the primary endpoint occurred significantly more frequently in patients with HF at baseline (HR of 1.81; CI: 1.08–3.04, *p* = 0.03) [30]. This suggests that further stratification by baseline HF status is needed to better understand the potentially different outcomes within the treatment group. Akrami et al. (2021) also found that among patients with acute coronary syndrome (ACS), colchicine significantly reduced major adverse cardiac events (MACE) overall, including ACS, stroke, survival rate, and decompensated HF, with a cumulative incidence of 6.7% in the colchicine group versus 21.7% in placebo group (*p* = 0.001). The decompensated HF rate itself also trended more favorably for the colchicine group (HR of 1.93; CI: 1.71–2.18), supporting the potential of early colchicine intervention to favorably influence at-risk populations before progression to HF [31].

In a trial by Deftereos et al. (2014) involving patients with stable HF with EF < 40% and average NYHA class of 2.4 ± 0.5 (NYHA IV excluded), colchicine led to significantly greater reductions in left ventricular end-diastolic and end-systolic diameters. Despite overall hsCRP reduction in the colchicine group (mean of −5.1 mg/l, *p* < 0.001), lack of improvement in the primary endpoint of NYHA class reduction and continuously elevated post-treatment hsCRP level (mean of 5.5 mg/l) suggest an inadequate inflammatory response reduction to drive clinical benefit [32].

The COLICA (Colchicine in Acute Heart Failure) trial observed that colchicine significantly reduced inflammatory markers CRP (−70.8% in colchicine group vs −51.1% in placebo; ratio of change of 0.60, *p* < 0.01) and IL-6 (−49.9% in colchicine group vs −30.1% in placebo; ratio of change of 0.72, *p* = 0.019) in patients with acute HF (NYHA Class II-IV). However, there was no clinical benefit and no between-group differences in NT-proBNP levels, NYHA class, or HHF and/or mortality risks [33]. The inclusion of predominantly late-stage NYHA Class III and IV patients and subsequent results underscore the potentially diminishing clinical impact of colchicine in later HF stages compared to the more optimal results observed in trials examining earlier stages of the disease. Moreover, COLICA did not select patients with elevated baseline inflammatory markers, which may explain the lack of clinical benefit despite the significantly reduced hsCRP and IL-6.

These trials investigating colchicine in cardiovascular disease and HF have several strengths and limitations. One of the main strengths of COLCOT is its large sample size of 4,745 patients. However, baseline hsCRP was only measured in a small subset of 207 patients, limiting insight into the degree of immune modulation and how this correlated to clinical outcomes. Both the COLICA trial and the study led by Akrami et. al (2021) were limited by small sample size, though the latter revealed improved clinical endpoints. COLICA also had high therapy discontinuation rates in both groups (~ 25% of participants) and an older study population (median age 75 years) compared to other trials, which may limit generalizability. The trial by Akrami et. al also did not measure inflammatory biomarker levels, thus data on the correlation between anti-inflammation and MACE reduction remains unknown. Overall, these limitations highlight the importance of selecting patients most likely to benefit from anti-inflammatory therapy and highlight the need for future studies on colchicine to stratify by HF phenotype and disease stage.

### Anti-myeloperoxidase (MPO) therapy

Myeloperoxidase (MPO) is a leukocyte-derived enzyme that links reactive oxygen species to inflammation, making it a potential target for cardiovascular disease. In the SATELLITE trial, mitiperstat, an MPO inhibitor, was evaluated in patients with NYHA class II-IV symptoms, specifically HFpEF or heart failure with mildly reduced ejection fraction (HFmrEF). While the SATELLITE trial was terminated prematurely due to the COVID-19 pandemic, it demonstrated a significant reduction in its primary endpoint of MPO activity by 75% (*p* = 0.001). Additionally, there was a trend towards improvement in quality-of-life, depicted by the + 6.283 point least squares mean difference (CI: 0.461–13.027, *p* = 0.067) in the Kansas City Cardiomyopathy Questionnaire (KCCQ) summary score [34].

The ENDEAVOR trial also further studied the effects of mitiperstat in symptomatic NYHA Class II-IV HFpEF or HFmrEF patients. Although the full report of ENDEAVOR has yet to be published at the time of this review, the preliminary results, presented at the AHA Scientific Sessions 2024, revealed no improvements in the primary outcomes of baseline KCCQ scores and 6MWD at 16 weeks among mitiperstat-treated patients (2.5 mg and 5 mg groups combined) compared to placebo [36]. Secondary outcomes, including KCCQ and 6MWD at 24 and 48 weeks, MACE, HF hospitalizations, NT-proBNP, inflammatory markers (hsCRP, IL-6), and echocardiogram parameters, also showed no differences among groups [36]. The lack of anti-inflammatory effects could also explain the absence of clinical benefits in these participants.

In both the SATELLITE and ENDEAVOR trials, the absence of baseline NYHA Class distribution limits assessment of whether disease stage influenced treatment response. Additionally, SATELLITE enrolled fewer participants than anticipated, resulting in exploratory rather than inferential statistical analyses and did not measure inflammatory markers besides MPO activity. The ENDEAVOR trial also did not recruit patients with elevated systemic inflammation at baseline, as indicated by hsCRP or increased MPO-specific activity, which could potentially account for the lack of therapeutic benefit observed with mitiperstat in this patient population. Nonetheless, given that these trials represent some of the first investigations into MPO inhibition in HF, its possible role as a target for future HF therapeutics remains an open question under investigation.

### Non-specific immunomodulation therapy

Non-specific immunomodulation therapies (IMT), such as intravenous immunoglobulins (IVIG), influence the concentrations of cytokines and its modulators. Gullestad et. al (2001) studied IVIG in HF patients with LVEF < 40% and NYHA class II-III (NYHA 2.6 ± 0.1) symptoms. IVIG significantly induced an anti-inflammatory state compared to baseline, evidenced by a 57% increase in IL-1Ra (*p* < 0.001), 65% increase in IL-10 (*p* < 0.001), and a 37% reduction in TNF-α/soluble TNF receptors ratio (*p* < 0.01). Interestingly, the IVIG group also had a significant increase in LVEF (*p* < 0.01) and 24% reduction in N-terminal pro-atrial natriuretic peptide (NT-pro-ANP) levels (*p* < 0.001) from baseline, while the placebo group had no differences [37]. However, within the IVIG group, participants with markedly reduced LVEF and longer duration of HF symptoms did not experience LVEF improvement, possibly representing a subgroup of chronic HF with irreversible myocardial damage due to long-standing disease. These findings suggest that IVIG effectively induces an anti-inflammatory state and potentially improves hemodynamic parameters in selected HF patient populations, particularly those without advanced, irreversible cardiac remodeling.

In the ACCLAIM trial, Celacade, a device-based IMT that treats autologous blood ex vivo with oxidative stress and UV light before reinjection, was evaluated in HF patients (predominantly NYHA Class II-III with only 4% NYHA IV). Compared to placebo, the IMT group saw a significant improvement in quality of life, based on the −6.9 score change (*p* = 0.04) in the Minnesota Living with Heart Failure Questionnaire (MLHFQ). Although the primary endpoint of death or cardiovascular hospitalization did not differ significantly between groups, stratification by NYHA class revealed that patients with NYHA II symptoms who received IMT experienced significantly better primary endpoint outcomes than their placebo counterpart (HR 0.61; CI: 0.46–0.80; *p* = 0.0003) [38].

The CORTAHF (Effect of Short-Term Prednisone Therapy on CRP Change in Emergency Department Patients with Acute Heart Failure and Elevated Inflammatory Markers) trial showed that 7 days of prednisone therapy significantly reduced hsCRP (adjusted geometric mean ratio of 0.30 in prednisone group vs 0.40 in placebo, *p* = 0.0498) in acute HF patients with elevated hsCRP at baseline. Prednisone also resulted in a decreased 90-day risk of worsening HF, HF readmission, or death (10.4%) compared to usual care (30.8%) (HR of 0.31; CI: 0.11–0.86, *p* = 0.016). Quality of life, as measured by the EuroQol 5-Dimension 5-Level analogue scale, also improved more in the prednisone group (least squares mean difference 2.57, 95% CI 0.12–5.01 points, *p* = 0.040) [39]. In CORTAHF, the patients had the following baseline NYHA class II-IV composition: II (16%), III (73%), and IV (11%). However, since the results were not stratified by NYHA class, it remains unclear whether disease severity influenced the observed benefits. As a pilot study and one of the first to investigate corticosteroids in HF, these findings warrant further investigation in larger trials to better define patient selection, long-term safety, and the potential role of steroids in HF.

Together, these exploratory studies highlight the potential benefit of non-specific IMT, particularly in patients with earlier-stage HF or with acute HF, where anti-inflammatory treatments may offer improvements in both hemodynamic parameters and quality of life. However, several limitations do exist within these trials. The study led by Gullestad et. al (2001) and CORTAHF had small number of participants. The investigators assessing outcomes in CORTAHF were also not blinded. However, the risk of bias introduction was mitigated by the use of objective endpoints, such as hsCRP and readmission rates. The ACCLAIM trial, despite its large sample size, failed to meet its primary endpoint, possibly due to inadequate inflammation suppression, as depicted by unchanged hsCRP levels. However, its promising results in NYHA II participants, as well as findings from IVIG and corticosteroid studies, underscore the need for larger trials with more precise HF patient stratification and selection.

## Future directions

Although inflammation is recognized as a key contributor to both the onset and progression of HF, clinical trials targeting inflammatory pathways have yielded inconsistent outcomes. This is likely due to heterogeneity in the disease stage of patients included in the trials as well as timing of intervention. Emerging evidence suggests that the HF stage at which anti-inflammatory therapies are administered appears to influence outcomes, with early intervention in at-risk populations showing improved clinical outcomes. Careful stratification of patients based on the HF stage may help identify subpopulations who may benefit from therapies early in disease course or even for prevention of symptom development. For example, in patients with recent myocardial infarction or those with elevated inflammatory markers without overt HF symptoms, early intervention may yield more favorable outcomes than treatments initiated in later stages when irreversible myocardial damage has occurred.

Current clinical trial designs often fail to capture the temporal dynamics of inflammation across HF stages. Inflammation in HF is dynamic and evolves with disease progression and fluctuates in response to acute events, such as myocardial injury and decompensation. Therefore, further mechanistic studies are needed to delineate how inflammatory activity evolves over time and to identify optimal therapeutic windows for intervention. We propose that measuring inflammation at multiple time points throughout the HF course could inform more precise timing of anti-inflammatory interventions. Treating patients during periods of heightened inflammatory activity may be more effective than treating chronic, low-grade inflammation that may not be driving disease progression. Additionally, pre-enrollment immune profiling using biomarkers to identify patients with biologically active inflammation could enhance patient selection, increasing the likelihood of therapeutic benefit. Serial biomarker assessments before, during, and after therapy may further refine the identification of optimal treatment windows. Additionally, current clinical trials classify patients based on symptom severity. Stratification using disease stage, possibly with imaging and/or biomarkers, may also help in determination of timing of intervention.

Future studies should also focus on identifying biomarkers that can be used to ensure adequate suppression of inflammation with anti-inflammatory therapies. Further mechanistic studies may also lead to identification of inflammatory mediators that are specific to HF, which can in turn lead to development of more targeted anti-inflammatory therapies with less side effects. Moreover, identifying biomarkers that accurately reflect inflammatory activity within myocardial tissue could further enhance the precision of targeted therapies. Restoring inflammation resolution pathways, such as specialized pro-resolving mediators and regulatory cytokines (i.e., IL-10), also represents a promising avenue. This may be especially crucial in developing therapies for more advanced disease stages where myocardial damage has already occurred.

Additionally, emerging data highlight the differential roles of various immune cell subsets, such as neutrophils, monocytes, and T cells, in HF progression. Targeting specific immune cell subsets could attenuate inflammation with lesser degree of immunosuppression. However, potential risks, such as increased infection susceptibility, highlight the need for long-term follow-up in clinical trials to assess both efficacy and safety. By pursuing these directions, future research may clarify the therapeutic potential of targeted anti- or pro-inflammatory strategies and pave the way for more personalized and effective HF treatments.

## Conclusion

Inflammation is a key driver in the pathophysiology and progression of HF, from early myocardial insult to ECM remodeling to extensive fibrosis and irreversible structural changes. Many of the trials targeting inflammatory pathways, such as TNF-α inhibitors, anti-IL-1 therapy, and IMT treatment, suggest that the myocardium is more amenable to anti-inflammatory therapy at earlier HF stages, while in advanced HF, treatment efficacy remains limited. For example, the ACCLAIM trial showed that IMT had better clinical outcomes in specific subgroups (NYHA II only) but not in the overall population that included NYHA II-IV. Moreover, individuals at risk for HF (coronary artery disease, hypertension, diabetes, obesity, and cardiomyopathy) may benefit from anti-inflammatory therapies even before development of symptoms to prevent progression into overt HF. The CANTOS trial demonstrated that IL-1β inhibition reduced cardiovascular events in post-MI patients who were at-risk for HF, but the benefits were less clear in patients who had an established HF history at baseline. Meanwhile, other RCTs have demonstrated inadequate inflammation control to see any of the anti-inflammatory benefits in the studied HF population. Despite these challenges, intervention, especially at earlier stages of HF in which substrates may be less irreversible, may be a promising strategy. We anticipate that inflammation-targeting therapies may yield more favorable outcomes at these stages, where the myocardium remains modifiable and the potential for therapeutic intervention is greater. Ultimately, as observational and experimental data continue to refine mechanistic targets, determining optimal HF substrates in which to deploy these targets will likewise be essential to inform clinical trials and the future of immune-targeted therapy for HF.

## Data Availability

No datasets were generated or analysed during the current study.
